# Love beyond gluten: self-esteem, illness identity, and social support in romantic rejection concerns among adolescents with celiac disease

**DOI:** 10.3389/fpsyg.2024.1335201

**Published:** 2024-05-20

**Authors:** Maor Shani, Maarten H. W. van Zalk

**Affiliations:** Department of Developmental Psychology, Institute for Psychology, Osnabrück University, Osnabrück, Germany

**Keywords:** romantic rejection, celiac disease, gluten-free, adolescence, illness identity, health-related quality of life

## Abstract

**Introduction:**

Fear of stigmatization, high perceived partner burden, or refraining from self-disclosure may manifest in romantic rejection concerns among adolescents with celiac disease (CD), potentially impacting their health-related quality of life (HRQOL). This study examined the prevalence, predictors, and consequences of romantic rejection concerns among adolescents and young adults with CD.

**Methods:**

A cross-sectional online survey was conducted among 165 German adolescents and young adults (aged 14–22) with self-reported CD. Participants completed measures of romantic rejection concerns, illness identity, self-esteem, peer support, and CD-specific HRQOL.

**Results:**

Participants reported moderate levels of concerns about the impact of CD on their romantic relationships, with no significant gender differences. Participants involved in romantic relationships expressed lower concerns of rejection, but similar preference for a “gluten-free partner.” Hierarchical regression analysis revealed that higher illness acceptance and peer support predicted lower rejection concerns. Significant interactions emerged between self-esteem and illness acceptance, and between self-esteem and peer support. Higher illness acceptance predicted fewer rejection worries only among those with high self-esteem, while peer support played a mitigating role only for those with low self-esteem. Romantic rejection concerns significantly predicted lower CD-specific HRQOL across all domains.

**Discussion:**

Anxieties about CD's impact on romantic relationships are prevalent among adolescents and may hinder their HRQOL. The findings highlight the complex interplay between self-esteem, illness identity, and social support in shaping romantic concerns. Targeted interventions focusing on peer support and fostering positive illness identity are recommended to alleviate rejection fears and improve HRQOL among youth with CD.

## 1 Introduction

“*I feel like a girl won't dig me if I'm overly picky ‘bout restaurants and the food that I order... she will think I'm nuts… I'm also concerned no one will ever want to marry me because they will be concerned that whatever I have will be passed down to our children... silly maybe but this is the type of stuff I dwell on*.”

(Anonymous post in Coping with Celiac Disease forum, Celiac and dating/relationships, 2008).

Adolescence, a crucial life phase noted for heightened sensation-seeking and peer orientation, is pivotal in molding beliefs, values, and aspirations (Dahl et al., 2018). It witnesses the onset of romantic relationships, key milestones that substantially sway social, emotional, and cognitive development (Collins et al., 2009). The inability to form such relationships can lead to diminished lifelong wellbeing and severe psychological impacts (Kansky and Allen, 2018; Schacter et al., 2019). Amplified by adolescence's cognitive malleability and sensitivity to social evaluation, romantic rejection further contributes to long-term relationship anxieties and hampers healthy sexual relationship progression (Glickman and La Greca, 2004).

These challenges are accentuated in adolescents with chronic illnesses who face developmental hurdles while grappling with their health conditions (Jordan et al., 2021). Individuals with chronic illnesses often cope with diminished autonomy, compromised social ties, feelings of alienation, potential depression and anxiety, and embarrassment linked to their condition (Juth et al., 2008; Pinquart and Shen, 2011; Arnone and Fitzsimons, 2012; Jordan et al., 2013; Schroeder and Mowen, 2014; Lambert and Keogh, 2015).

Despite substantial advances in understanding adolescents' romantic relationships and the psychological impacts of managing chronic illness, these research domains have seldom been combined. Consequently, the understanding of romantic relationships among young individuals with chronic illnesses remains limited. Prior studies primarily focused on those already in romantic or sexual relationships, neglecting the anxieties experienced in anticipation of intimate relationships (Jordan et al., 2021). Few studies have explored how coping with chronic conditions may affect youth's attitudes and emotions toward romantic relations.

This study addresses this gap by examining the antecedents and consequences of romantic rejection worries or concerns (used interchangeably) among focusing on adolescents and young adults with celiac disease (CD), a chronic, immune-mediated enteropathy managed by a lifelong gluten-free diet (GFD) that substantially influences quality of life (Lebwohl et al., 2018). Our study pursues answers to the following research questions: (1) How does living with CD affect adolescents' perceptions of romantic relationships and rejection fears? (2) Are there differences in the levels of romantic rejection worries between genders, and between adolescents in and not in a relationship? (3) What role can key factors in managing chronic conditions, namely illness identity and peer support, play in such romantic worries? (4) And finally, how do such concerns, if present, affect their health-related quality of life (HRQOL)? This work offers both theoretical insights into CD's impact on youth's anxieties, quality of life, and psychosocial health, and practical contributions for creating strategies to support youth with CD in managing negative emotions, strengthening resilience, and fostering positive attitudes toward relationships.

### 1.1 Romantic relationships and rejection concerns among adolescents with celiac disease

Adapting to CD and maintaining a GFD pose significant challenges for adolescents transitioning to independence, often leading to decreased adherence and increased mental health issues (Arnone and Fitzsimons, 2012; Schroeder and Mowen, 2014). The burden of adhering to dietary restrictions, managing social scenarios, and handling physical symptoms can amplify feelings of social exclusion, increase feelings of isolation and frustration, and reduce life quality during this pivotal period (Sverker et al., 2005; Rosén et al., 2011; de Lorenzo et al., 2012; Skjerning et al., 2014; Woolley et al., 2020).

Research on the impact of CD on romantic relationships has been scarce (Alley, 2015; Roy et al., 2016; Boon and Holmgren, 2019; Lebovits et al., 2022; Villafuerte-Gálvez and Silvester, 2022). Jordan et al. (2021) conducted a review and concluded that adolescents with chronic conditions desire romantic relationships similarly to their healthy peers but worry about rejection due to perceived unattractiveness caused by their disease. Adolescence with CD may fear negative reactions akin to those experienced by individuals with food allergies, including pressure for unhealthy behaviors (Daum, 2022). Behaviors necessary for managing CD may be seen as unattractive, creating barriers to forming relationships (Aloni et al., 2019). Stigma related to CD can also impact relationship initiation and maintenance, with self-concepts and social support systems often negatively affected (Schroeder and Mowen, 2014; Ruddy and Taft, 2022).

Moreover, shame associated with chronic illnesses can impact the quality of social relationships and mental health, with fears of receiving compassion undermining the onset of relationships (Trindade et al., 2018). Self-disclosure of CD in an intimate context may also be a source of anxiety, contributing to the complexity of romantic engagements (Defenbaugh, 2013). A recent survey found that < $\frac{1}{2}$ of CD patients communicated their diagnosis before their first date, and only a minority included it on their online dating profile (Villafuerte-Gálvez and Silvester, 2022). Lastly, adolescents with CD may worry about the perceived burden they might place on a potential partner due to their disease and dietary restrictions. These anxieties may lead to unfavorable dating experiences, as recently reported in the U.S. (Lebovits et al., 2022) and Columbia (Villafuerte-Gálvez and Silvester, 2022). Combined with more generalized socioemotional anxieties, they can lead to heightened concerns about romantic rejection, influencing their dating experiences and potentially diminishing their romantic involvement over time.^1^

### 1.2 The role of peer support in rejection concerns

Should adolescents with CD harbor fears of romantic rejection due to their condition, we may ask which social and psychological factors, general or illness-related, could guard against such concerns and mitigate their potential negative impact on psychosocial health. This study targets two psychological resources recently demonstrated to bolster coping strategies for chronic illnesses, including CD.

First, we consider the role of *peer social support*, a vital factor in buffering stress emanating from romantic rejection anxieties and adverse perceptions of romance and dating due to CD (Letkiewicz et al., 2023). Instrumental and emotional support, comprising practical assistance, decision-making, empathy, and compassion, can mitigate stressful events in chronic conditions, enhancing psychosocial adaptation (White et al., 2018; Lehardy and Fowers, 2020). Such support may also foster quality of life outcomes by nurturing feelings of connectedness and belonging (Helgeson, 2003). In the case of adolescents with CD, social support may be an effective coping strategy (Meijer et al., 2002; Köstlin et al., 2023). Such support was associated with better self-esteem, overall mental health, and lower feelings of guilt and being different (Sverker et al., 2005). Focusing on peer support, evidence indicates that peers, who are often in similar situations, are likely to provide effective social support in adolescents with CD (Köstlin et al., 2023). In general, peer support was associated with improved psychosocial functioning, mental health, reduced loneliness, increased social acceptance, and self-efficacy in various chronic conditions (Kulandaivelu and Kohut, 2021; Letkiewicz et al., 2023). Therefore, we posit that peer social support can significantly help adolescents with CD manage the stress of romantic rejection worries.

### 1.3 The role of positive illness identity in rejection concerns

A second potential buffer against negative socioemotional coping with CD in general and romantic rejection concerns in particular is illness identity, conceptualized as the way individuals integrate their illness experiences into their broader self-concept (Oris et al., 2016). Illness identity can be divided into four distinct domains: *engulfment* is characterized by the disease being a dominant aspect of a person's identity, often overwhelming other aspects of self-perception; *rejection* occurs when individuals resist associating themselves with the disease, often refusing to incorporate it into their self-identity; *acceptance* represents a balanced integration of the disease into one's identity without allowing it to overshadow overall self-concept; and *enrichment* is characterized by a positive transformation of self and a sense of personal growth following the illness experience (Oris et al., 2016).

In this study we focus on the two positive domains of illness identity, namely acceptance and enrichment. Raymaekers et al. (2020) demonstrated that a maladaptive illness identity predicted difficulties in forming healthy peer relationships among adolescents with diabetes, while high acceptance predicted healthy peer interactions. This suggests the significant role social context plays in molding illness identity. Inadequate acceptance of CD into one's identity may stem from the fear of excessive focus on the illness by others, leading to poor treatment adherence and maladjustment. Hence, concerns about romantic rejection may be related to distancing one's illness from identity, that is, low illness acceptance.

Enrichment is another adaptive and resilient facet of positive illness identity. When combined with peer support—given research indicates participation in online peer groups bolsters individual and collective identity development (Lehardy and Fowers, 2020)—enrichment may play a particularly adaptive role in managing CD-related stress, and therefore inversely associated with romantic rejection concerns.

### 1.4 The role of global self-esteem in rejection concerns

In understanding romantic rejection concerns it should be important also to consider personal psychological traits related to self-evaluation, of which self-esteem is particularly salient. Global self-esteem is defined as the “positive or negative attitude toward a particular object, namely, the self” (Rosenberg, 1965, p. 30). During adolescence, perceptions of physical and romantic attractiveness significantly influence self-worth, with high self-esteem predicting successful romantic engagement (Luciano and Orth, 2017). Conversely, low self-esteem is linked to fears of social rejection, emotional vulnerability in relationships (Leary and Baumeister, 2000), and an anxious anticipation or excessive response to social rejection (Khoshkam et al., 2012).

The ability to maintain romantic relationships in chronically ill youth was found to be positively associated with self-esteem (Balch, 2014; Jordan et al., 2021). Building upon studies showing self-esteem's moderating effects on rejection responses (Sommer and Baumeister, 2002; Waller and MacDonald, 2010) and psychological stress in youth (Kong et al., 2013), we aimed to elucidate the interaction between global self-esteem, peer support, and illness identity in relation to romantic rejection. Specifically, we explored whether peer support and illness identity offer comparable protection against romantic rejection concerns across different self-esteem levels.

### 1.5 The potential contribution of romantic rejection concerns to HRQOL

Finally, we aim to examine romantic rejection concerns in the context of HRQOL. HRQOL is defined as “the extent to which one's usual or expected physical, emotional, and social wellbeing are affected by a medical condition or its treatment” (Cella, 1995, p. 73). The recent shift from generic to CD-specific HRQOL measures signifies the importance of unique CD experiences in determining quality of life (Burger et al., 2019). Accordingly, CD-specific measures of HRQOL capture negative emotions, such as embarrassment and shame, the balance between social participation and isolation, and future concerns related to CD (Jordan et al., 2013). Such measures have shown a lower quality of life compared to generic instruments, highlighting their superiority for comparative group analysis (Barrio and Cilleruelo, 2022).

However, the specific facet of romantic relationships is often overlooked in these evaluations. Food-centered social behaviors significantly contribute to the social burden of CD, potentially leading to negative HRQOL outcomes (Lebovits et al., 2022). Hullmann et al. (2012) found that heightened anxiety, fear of negative peer evaluation, and lower rates of romantic relationships among adolescents with food allergies predicted poorer HRQOL and diminished social functioning. Examining the predictive role of romantic rejection concerns in CD-specific HRQOL could guide healthcare providers and practitioners in their evaluations, highlighting the importance of addressing these issues in patient care.

## 2 Method

In this cross-sectional study, we sourced a convenience sample of German adolescents and young adults (ages 14–22) diagnosed with CD. Given the relatively low prevalence of Celiac Disease (CD) within this demographic, achieving representative sampling was deemed impractical. Consequently, participant recruitment was facilitated through the Youth Committee of the German Celiac Society (DZG), utilizing mailing lists, social media platforms (Facebook and Instagram), and an advertisement in the society's newspaper. This advertisement invited adolescents and young adults to partake in an online survey titled “Experiences of Youth with Celiac,” accessible via a link or QR code provided in the recruitment ad. No financial or other forms of incentives were offered. Evaluating the response rate was not feasible; however, based on information provided by the DZG, it is estimated that a few 100 potential participants were exposed to the recruitment calls across the various media outlets. The participants completed the online questionnaires during February and March 2019. Ethical approval was granted by the first author's institute. For those under 18, parental permission was required in addition to their informed consent. All data and analysis codes are accessible at: https://osf.io/xsdn2/.

### 2.1 Participants and procedure

Using G^*^Power, an a priori power analysis indicated a required sample size of 126 to detect a medium-sized bivariate correlation (*r* = 0.30) at α = 0.05 and 1 – β = 0.80. A larger sample was eventually obtained, *N* = 165. Power analysis for moderation effects is detailed in the Results section (Baranger et al., 2022). Inclusion criteria encompassed German-speaking individuals aged 14–22 with self-reported CD. Participants were not asked for medical details related to their diagnosis. An online questionnaire captured adolescents' experiences coping with CD as part of a broader study (see BLINDED FOR REVIEW).

Sample demographic and illness-related characteristics are provided in the Supplementary Table 1. The sample comprised 134 females, 28 males, and three undisclosed gender, averaging 17.18 years (*SD* = 2.50). The average duration of illness was 7.69 years (*SD* = 5.04). Roughly 40.6% reported severe gluten exposure symptoms, 31.5% moderate, and 15.8% none. Thirty-eight percentage indicated other medical conditions, 24% mild food allergies, and 17% serious allergies. About two-thirds (*n* = 114) were not in a relationship. Those in a relationship were older on average (*M* = 17.92, *SD* = 2.62) than non-relationship participants (*M* = 16.84, *SD* = 2.38), *p* = 0.010. We retained all participants regardless of relationship status, which may allow us to examine whether worries about anticipated romantic rejection may also characterize adolescents who are romantically involved. In addition, we examined differences in rejection concerns by reported relationship status.

### 2.2 Measures

The instruments used were either originally in German, obtained as pre-validated German translations, or meticulously translated into German through a translation-back translation process by bilingual researchers proficient in both English and German. For multi-item scales, scores were computed by averaging all items, post re-scoring of reverse-worded items, where required, to formulate scales. Unless mentioned otherwise, all items were rated on a scale ranging from 1 (*strongly disagree*) to 5 (*strongly agree*).

#### 2.2.1 Attitudes toward romantic relations and CD

We initially constructed a 12-item measure based on a focus group with adolescent DZG volunteers discussing CD coping difficulties, including worries about rejection due their illness, worries about disclosure of their illness to potential partners, and attitudes toward having gluten-free partners. After cognitive pretesting, this was reduced to eight items, with examples such as “If I date someone, they should know nothing about my celiac disease,” and “I sometimes worry that no one wants to be with me because of my celiac disease.” Following Principal Component Analysis with Varimax rotation, two factors explaining 56.1% of the variance emerged, but two items with low loadings were discarded (see detailed results in the Supplementary Table 2). Two scales were computed: one four-item scale on romantic rejection concerns (α = 0.94), and another two-item scale on romantic preferences (*r* = 0.34).

#### 2.2.2 Peer support

Perceived peer support, reflecting individuals' perceived understanding and sympathy from friends regarding their CD, was measured with five items adapted from existing social support measures (Procidano and Heller, 1983; Zimet et al., 1988). These items gauged participants' perceptions of peer support in managing their disease and diet (e.g., “I enjoy talking with my friends about my celiac,” and “I feel comfortable eating together with my friends,” α = 0.67). Higher scores indicated greater perceived peer support.

#### 2.2.3 Illness identity (acceptance and enrichment)

Adaptive identity integration was measured using two domains from the Illness Identity Questionnaire (Oris et al., 2016): a *5-item acceptance* scale and a 7-item *enrichment* scale, with example items such as “My celiac is a part of who I am,” and “Because of my illness, I realize what is really important in life.” This questionnaire, originally developed for type-1 diabetes patients, was adapted to CD context. We chose to focus on the adaptive dimensions of illness identity for several reasons. First, the extant literature, applying Oris et al.'s (2016) framework, demonstrates that adaptive and maladaptive illness identity domains exhibit inverse correlation patterns with physiological and psychological outcomes, as well as overall HRQoL (e.g., Meyer and Lamash, 2021; Rassart et al., 2023; see review: Shneider et al., 2023). This suggests that an understanding of the relationship between illness identity and outcomes may be adequately achieved by examining the strength of integrative domains. Second, among adolescents and young adults, acceptance and enrichment have been shown to correlate more significantly with adaptive identity trajectories compared to engulfment and rejection (Vanderhaegen et al., 2024). Finally, prioritizing integrative aspects of illness identity proved beneficial in minimizing questionnaire length and cognitive burden on participants, particularly adolescents. A Confirmatory Factor Analysis (CFA) validated the measure, showing satisfactory model fit according to common thresholds (Hu and Bentler, 1999), after adding covariates between two pairs of item errors, $\chi_{\left(51\right)}^{2}$ = 74.652, *p* < 0.05, CFI = 0.970, TLI = 0.962, RMSEA = 0.057 (for detailed model results see Supplementary Table 3). Two scale scores were computed, acceptance (α = 0.84) and enrichment (α = 0.88), with higher scores indicating a more positive illness identity.

#### 2.2.4 Self-esteem

Self-esteem was measured using a single-item assessment, as outlined by Robins et al. (2001). Validated against the Rosenberg Self-Esteem Scale (Rosenberg, 1965), this method offers a reliable estimation of global self-esteem. Participants rated their agreement with “I have high self-esteem” on a 7-point Likert scale, which ranges from 1 (*strongly disagree*) to 7 (*strongly agree*). Though single-item measures can be less reliable and sensitive (Diamantopoulos et al., 2012), this method was chosen for efficiency within a larger assessment battery. Its brevity minimizes participant burden and fatigue, enhancing data quality.

#### 2.2.5 Celiac disease-specific health-related quality of life

We used the CDPQOL 13 to 18, a pediatric CD-specific HRQOL tool that focuses on stigmatization and social participation difficulties (Jordan et al., 2013). Developed from US focus groups and expert interviews, its relevance extends to young European patients. Participants rate their experiences over the past month on a 17-item scale from 0 (*never*) to 4 (*almost always*). The instrument defines four domains: Social (self-esteem, being a burden), Uncertainty (future worries), Isolation (feeling different), and Limitations (negative emotions about avoidance). A CFA validated this structure, yielding an acceptable model fit, $\chi_{\left(110\right)}^{2}$ = 151.204, *p* < 0.01, CFI = 0.954, TLI = 0.944, RMSEA = 0.050, with sufficiently reliable Cronbach's α scores (Social: α = 0.84; Uncertainty: α = 0.61; Isolation: α = 0.72; and Limitations: α = 0.63). Although alpha values of uncertainty and limitations were lower than 0.70, recent scholarship posits that for scales with a limited number of items, a threshold of 0.60 may suffice to denote adequate reliability, given the potential bias introduced by having small number of items (Taber, 2018; Hair, 2019). Moreover, although prior research using the CDPQOL did not report reliability indices, we opted to retain the original measure without modifications to ensure consistency and facilitate comparative analysis with prior and future studies. In addition, to facilitate comparability, domain scores are computed as item averages, reversed, and scaled 0–100, with higher scores indicating better HRQOL.

#### 2.2.6 Demographics and CD variables

We gathered demographic data including age, gender, relationship status, and socioeconomic status (SES) gauged by participants on a 1–5 scale for family income level. We also evaluated participants' years living with CD and their adherence to the GFD. Adherence was determined by averaging responses to two items concerning strictness and frequency of gluten consumption, rated on a 1–10 scale (*r* = 0.670, *p* < 0.001).

### 2.3 Data analysis overview

Analyses were performed using R software version 4.1.1. Missing data, accounting for up to 4% per variable, were listwise deleted as Little's MCAR test was insignificant, $\chi_{\left(133\right)}^{2}$ = 141.026, *p* = 0.300. Initial analysis included descriptive statistics and independent-sample *T*-tests across gender and relationship status. Pearson correlations were used to examine bivariate relationships.

We applied hierarchical linear regression in three steps to determine the unique and joint contribution of predictors to romantic rejection concerns. First, we entered demographic and illness-related variables, followed by self-esteem, illness identity (acceptance and enrichment dimensions), and peer support in the second step. Lastly, two-way interactions between self-esteem, illness identity domains, and peer support were added. Simple slopes analyses followed significant two-way interactions. A second regression set predicted the CDPQOL dimensions and overall CDPQOL from demographic, illness related, and psychological variables, including romantic rejection concerns. In regression analysis, we used the lm function regression fitting and the jtools package, which provided robust estimates of significance. Multicollinearity was assessed using VIF values (< 5). Significance was determined at the *p* < 0.05 level using two-tailed tests.

## 3 Results

### 3.1 Prevalence of romantic rejection worries and preferences and comparison by gender and relationship status

Distribution of responses shows 20–33% of participants expressed concerns about CD's impact on their romantic lives (see Supplementary Figure 1). Around 30% worry their disease might deter potential partners, with about one-fifth concerned it may preclude dating entirely. Only 13% preferred concealing their disease, while over a quarter preferred a partner also with CD or following a GFD. Overall, these findings suggest that worries about CD-related romantic rejection are not negligible among a normative sample of German youth with CD, and that such concerns are relatively widespread.

Supplementary Table 4 depicts means and standard deviations for romantic concerns and preferences items. The average perception of CD as a limiting factor in romantic relationships was modest (*M* = 2.22, *SD* = 1.05). Boys displayed lower indifference toward having a celiac partner than girls [*t*_(159)_ = 2.19, *p* = 0.030, *d* = 0.46]. However, no other significant gender differences existed in romantic concerns or preferences items. Non-relationship participants showed more worry about potential partners not dating them [*t*_(162)_ = 2.70, *p* = 0.008, *d* = 0.46], and about difficulty in finding a partner due to CD [*t*_(163)_ = 2.55, *p* = 0.012, *d* = 0.43], resulting in higher average romantic rejection worries [*t*_(162)_ = 2.34, *p* = 0.020, *d* = 0.40]. No significant differences were found in preferences for celiac partners or in main variables between relationship status subgroups.

### 3.2 Correlational analysis

Bivariate Pearson correlations between the study's variables are presented in Table 1. A stronger preference for a partner with CD was found to correlate with higher romantic rejection worries. Moreover, higher rejection worries associated with lower self-esteem and CDPQOL across all domains and overall. These worries were also associated with lower social support and lower acceptance of illness as part of one's identity. However, they did not significantly correlate with identity enrichment or adherence to the GFD. Concerning preference for a partner with CD, weaker-to-moderate associations were discovered between a higher preference for such a partner and lower CDPQOL, specifically in terms of uncertainty, isolation, and limitation aspects, as well as overall quality of life. This preference was weakly linked to lower social support but had no significant connection to illness identity or self-esteem.

**Table 1:** Means, standard deviations, and Pearson correlations between the study's main variables.

**Variable**	***M***	***SD***	**1**	**2**	**3**	**4**	**5**	**6**	**7**	**8**	**9**	**10**	**11**
1. Romantic concerns	2.29	1.29											
2. Romantic preference	2.19	1.05	0.36^**^										
3. Self-esteem	3.20	1.31	−0.24^**^	−0.06									
4. CDPQOL-social	58.03	21.33	−0.41^**^	−0.15	0.37^**^								
5. CDPQOL-uncertainty	69.41	25.59	−0.39^**^	−0.21^**^	0.36^**^	0.58^**^							
6. CDPQOL-isolation	67.94	21.88	−0.35^**^	−0.16^*^	0.34^**^	0.62^**^	0.62^**^						
7. CDPQOL-limitation	60.24	25.62	−0.44^**^	−0.20^**^	0.44^**^	0.69^**^	0.50^**^	0.64^**^					
8. CDPQOL-overall	62.91	19.46	−0.45^**^	−0.23^**^	0.43^**^	0.90^**^	0.78^**^	0.82^**^	0.82^**^				
9. Peer support	2.97	0.77	−0.31^**^	−0.16^*^	0.36^**^	0.52^**^	0.41^**^	0.46^**^	0.52^**^	0.56^**^			
10. Identity acceptance	4.01	0.83	−0.25^**^	−0.11	0.31^**^	0.33^**^	0.32^**^	0.41^**^	0.22^**^	0.36^**^	0.42^**^		
11. Identity enrichment	2.82	0.98	0.01	0.06	0.23^**^	0.03	0.11	0.19^*^	0.06	0.08	0.15	0.34^**^	
12. GFD adherence	9.55	0.97	−0.03	−0.02	0.14	0.09	0.22^**^	0.25^**^	0.00	0.16^*^	0.15^*^	0.35^**^	0.12

CDPQOL, Celiac Disease Pediatric Quality of Life; GFD, Gluten-free diet.

^*^p < 0.05.

^**^p < 0.01.

### 3.3 The role of self-esteem, illness identity, and peer support in predicting romantic rejection worries

Hierarchical linear regression was utilized to predict romantic rejection worries, as shown in Table 2. The initial model incorporating socio-demographic and CD-related variables accounted for 6.3% of the variance, with relationship status being the only significant predictor, *B* = −0.533, *p* = 0.028. Self-esteem, illness identity dimensions, and peer social support were added in the second model, accounting for 18.2% of the variance, Δ*R*^2^ = 0.119, *p* = 0.002. Relationship status persisted as a predictor, *B* = −0.501, *p* = 0.031, with higher illness acceptance, *B* = −0.371, *p* = 0.032, and peer social support, *B* = −0.364, *p* = 0.026, also significantly predicting lower worries. The final model, incorporating two-way interactions, accounted for 28.6% of the variance, Δ*R*^2^ = 0.104, *p* < 0.001, with relationship status remaining a significant predictor, *B* = −0.484, *p* = 0.028. Two significant interactions emerged: between self-esteem and illness acceptance; and self-esteem and peer support. The analysis had sufficient power (>0.77) to detect each interaction considering the sample size and the correlation matrix (Baranger et al., 2022).

**Table 2:** Results of hierarchical linear regression predicting romantic rejection concerns.

**Predictors**	**Model 1**				**Model 2**				**Model 3**			
	***B***	***SE***	**β**	***p***	***B***	***SE***	**β**	***p***	***B***	***SE***	**β**	***p***
(Intercept)	4.874	1.449	0.000	**0.001**	4.579	1.389	0.000	**0.001**	5.082	1.605	−0.040	**0.002**
Age	−0.005	0.046	−0.010	0.907	0.019	0.044	0.036	0.673	0.021	0.042	0.040	0.620
1 = male	0.113	0.292	0.033	0.700	0.108	0.278	0.032	0.698	0.207	0.265	0.061	0.437
SES	−0.333	0.179	−0.159	0.066	−0.201	0.177	−0.096	0.258	−0.278	0.170	−0.132	0.104
Years with CD	−0.008	0.022	−0.031	0.722	0.013	0.023	0.051	0.570	0.020	0.022	0.079	0.353
1 = In relationship	−0.533	0.241	−0.191	**0.028**	−0.501	0.230	−0.179	**0.031**	−0.484	0.218	−0.173	**0.028**
GFD adherence	−0.122	0.109	−0.096	0.265	0.049	0.114	0.039	0.668	−0.029	0.111	−0.023	0.795
Self-esteem					−0.058	0.095	−0.057	0.542	0.130	0.401	0.040	0.746
Illness identity: acceptance					−0.371	0.171	−0.234	**0.032**	0.834	0.364	−0.317	**0.024**
Illness identity: enrichment					0.152	0.117	0.114	0.198	−0.237	0.298	0.155	0.427
Peer support					−0.364	0.162	−0.217	**0.026**	−1.616	0.396	−0.201	**< 0.001**
Self-esteem X peer support									0.386	0.111	0.295	**0.001**
Self-esteem X illness identity: acceptance									−0.404	0.110	−0.327	**< 0.001**
Self-esteem X illness identity: enrichment									0.134	0.084	0.129	0.113
Observations	139				139				139			
*R*^2^/*R*^2^ adjusted	0.063/0.021				0.182/0.118				0.286/0.211			

GDF, Gluten-free diet. *P* values of significant predictors (*p* < 0.05) appear in bold.

**Figure 1:**
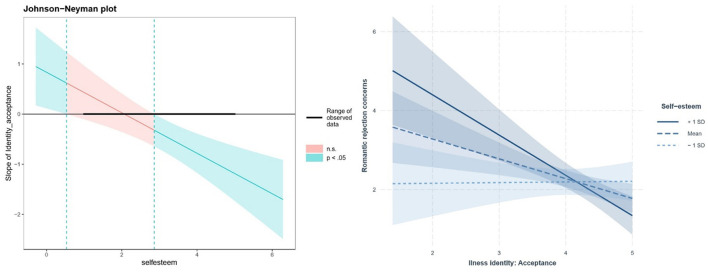
Interaction of self-esteem and illness identity acceptance: simple slope analysis. Hierarchical linear regression revealed a significant interaction between self-esteem and illness acceptance in predicting romantic rejection concerns, *B* = −0.404, *p* < 0.001. The Johnson-Neyman regions of significance **(left panel)** and simple slopes of illness acceptance for low (−1 *SD*), mean, and high (+1 *SD*) self-esteem **(right panel)** are depicted above.

**Figure 2:**
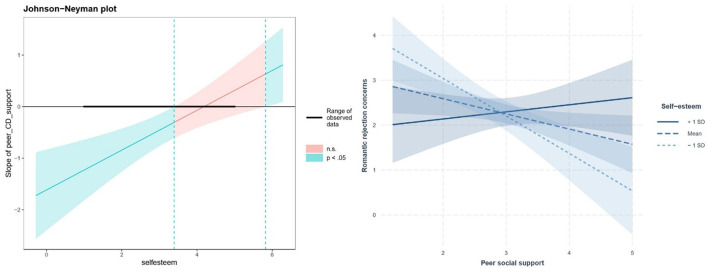
Interaction of self-esteem and peer support: simple slope analysis. A significant interaction between self-esteem and peer support in predicting romantic rejection concerns was found through hierarchical linear regression, *B* = 0.386, *p* = 0.001. The Johnson-Neyman regions of significance **(left panel)** and simple slopes of peer support for low (−1 *SD*), mean, and high (+1 *SD*) self-esteem **(right panel)** are displayed above.

The first interaction (depicted on Figure 1) involved self-esteem and illness acceptance in forecasting romantic rejection worries, *B* = −0.404, *p* < 0.001. Analysis clarified this interaction, revealing that the effect of illness acceptance was only significant with high (>2.863) or extremely low self-esteem (< 0.533). For average or high (+1 SD) self-esteem, higher illness acceptance predicted fewer romantic worries, *B* = −0.501, *p* = 0.003 and *B* = −1.020, *p* < 0.001, respectively. When self-esteem was low (−1 SD), the slope for identity acceptance was insignificant, *B* = 0.017, *SE* = 0.192, *p* = 0.930. The second interaction (depicted on Figure 2) occurred between self-esteem and peer support, *B* = 0.386, *p* = 0.001. Peer support only impacted romantic worries at lower self-esteem levels (< 3.394). For those with low or average self-esteem, increased peer support predicted fewer worries, *B* = −0.834, *p* < 0.001 and *B* = −0.338, *p* = 0.030, respectively. However, for high self-esteem individuals, peer support did not significantly affect romantic worries, *B* = 0.158, *p* = 0.455. In summary, the conditional effects analysis revealed that higher acceptance of illness identity predicted fewer worries about romantic rejection, but only for individuals with relatively high self-esteem. Conversely, higher peer social support predicted fewer worries, but only for those with relatively low self-esteem.

An additional exploratory analysis of the potential moderating role of gender is included in the Supplementary material but should be interpreted with caution (see Section 4.1).

### 3.4 The contribution of romantic rejection worries to celiac-disease quality of life

**Figure 3:**
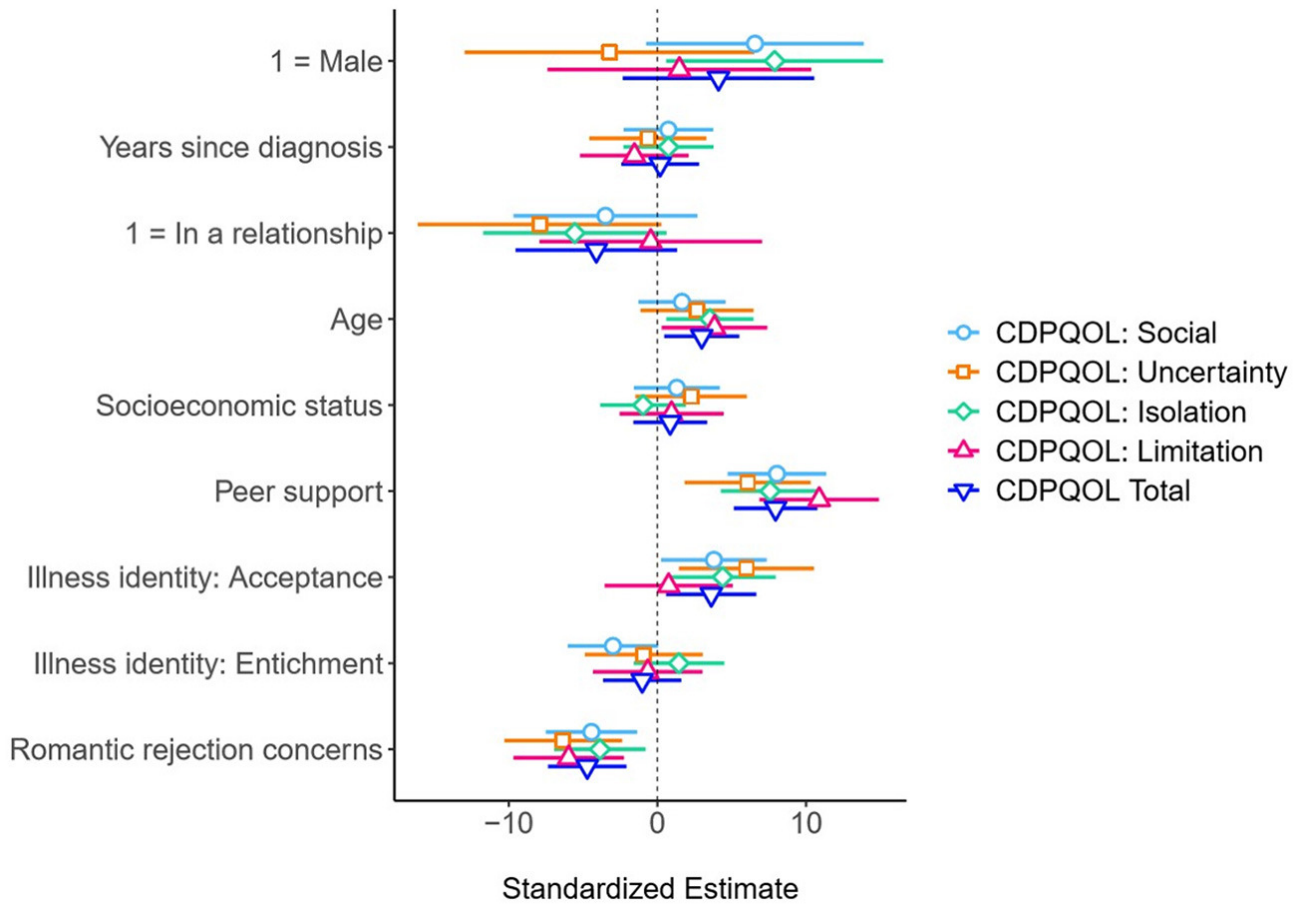
Standardized coefficients with 95% confidence intervals for linear regression models predicting CDPQOL. Shapes signify standardized regression coefficients, distinguished for each CDPQOL dimension. The horizontal lines flanking each shape represent 95% confidence intervals (CIs), derived from robust standard error. The absence of overlap between the CI and the vertical 0 indicates the significance of each coefficient (*p* < 0.05). For comprehensive numerical results, refer to Supplementary Table 5.

A final set of analysis was conducted to determine the added value of being concerned with romantic rejection in predicting CDPQOL, over and above other potential predictors Standardized regression coefficients for all models are depicted on Figure 3 and in details in Supplementary Table 5. According to the results, higher romantic rejection worries significantly predicted higher CDPQOL across all domains, beyond demographic and illness-related predictors (Social: *B* = −3.379, *SE* = 1.181, *p* = 0.005; Uncertainty: *B* = −4.841, *SE* = 1.529, *p* = 0.002; Isolation: *B* = −2.947, *SE* = 1.185, *p* = 0.014; Limitation: *B* = −4.545, *SE* = 1.431, *p* = 0.002; and overall CDPQOL: *B* = −3.604, *SE* = 1.019, *p* = 0.001).

## 4 Discussion

Considering the crucial role romantic relationships play in adolescents' development toward adulthood (Collins et al., 2009), it is important to investigate how its socio-emotional load manifests as anxiety regarding romantic reciprocity and potential partner rejection. This study provides an initial appraisal of romantic rejection concerns among CD-affected adolescents, exploring the associated adaptive and maladaptive psychological resources, which could guide counseling and interventions, with a particular focus on illness identity, social support, and self-esteem.

This study indicates that, beyond conventional attractiveness factors, anxieties related to CD and its treatment may impede the formation and maintenance of romantic relationships among affected youth. Our sample of German adolescents and young adults with CD showed moderate romantic rejection fears, with a substantial portion concerned about the impact of CD on their relationships and how partners might perceive their condition. Interestingly, only a few preferred to hide their illness, indicating a positive stride toward overcoming stigma often associated with chronic illness in romantic settings (Jordan et al., 2021). Fears of romantic rejection among CD patients may arise from internalized stigma, compassion fears linked with shame (Trindade et al., 2018), and the necessity for self-disclosure due to CD's invisible nature (Defenbaugh, 2013). This aligns with existing research showing how chronic illnesses can negatively impact relationships (Pinquart and Shen, 2011; Woolley et al., 2020), including reduced social participation and relationship quality among youth due to both CD and GFD adherence (Sverker et al., 2005; Rosén et al., 2011; Schroeder and Mowen, 2014; Skjerning et al., 2014).

The analysis further revealed a robust link between elevated romantic rejection concerns and enhanced CDPQOL across all domains, contributing to social anxiety, uncertainty, isolation, and disease-perceived limitations. These findings underscore the significance of romantic relationships during adolescence and early adulthood, and the psychosocial development threat posed by rejection (Glickman and La Greca, 2004; Kansky and Allen, 2018). They also highlight the necessity for tailored interventions addressing romantic rejection concerns, given their substantial impact on HRQOL. While studies often find comparable quality of life and social phobia levels between individuals with and without CD (Addolorato et al., 2008; de Lorenzo et al., 2012), our results highlight specific social anxieties in young CD patients, affecting their psychosocial functioning and life quality. This underlines the necessity of CD-specific instruments for evaluating psychosocial health (Burger et al., 2019; Barrio and Cilleruelo, 2022), which capture CD-impacted areas often overlooked by general instruments.

This research revealed that gender did not significantly impact concerns related to romantic rejection due to CD, with both genders expressing comparable levels of concern. Boys, however, exhibited less indifference to having a CD-afflicted partner. Despite CD's higher prevalence in females (Lebwohl et al., 2018), gendered experiences of CD might be comparable. Still, social experiences might differ qualitatively. For instance, due to associations of GFD with femininity, boys may fear rejection due to perceptions of femininity (Aloni et al., 2019). Further in-depth, qualitative research could clarify such nuances of romantic rejection fears. In contrast, we found that those with CD not currently in a relationship exhibited greater worry of potential partner rejection due to their condition compared to counterparts in relationships, indicating that involvement in relationship might alleviate such concerns. Nevertheless, the groups did not significantly differ in average concerns about concealing their condition to potential partners and in having general worries about rejection due to CD. Moreover, the study has not identified significant variation in other key variables based on relationship status, indicating a complex interplay of factors shaping romantic experiences for youth with CD, and the relevance of romantic concerns to adolescents based on their illness, even if they are romantically involved.

Our analysis demonstrated that being in a relationship, acceptance of illness, and higher peer support predict lower worries about romantic rejection in adolescents with CD. Most importantly, we identified two interaction effects: higher illness acceptance significantly predicts lower rejection concerns, but only for those with high self-esteem; On the other hand, higher peer support mitigated rejection worries, but only for those with low self-esteem. These findings are consistent with recent studies showing that self-esteem moderates psychosocial correlates of social rejection, and that adolescents with differential self-esteem react differently to perceived peer rejection (Waller and MacDonald, 2010). They also imply that positive psychosocial adjustment and supportive environments are key for those with low self-esteem to manage romantic anxieties. Adolescents with CD possessing low self-esteem may exhibit high rejection sensitivity (Khoshkam et al., 2012). They may therefore necessitate a supportive social milieu to mitigate anxieties concerning peer relationships. Conversely, those boasting higher self-esteem maintain their favorable self-views by focusing attention on their positive qualities, or self-enhancing (Bosson et al., 2003). As a result, there may experience diminished dependence on others and possess the capability to assimilate their illness positively into their identity, thereby bolstering their resilience and fostering self-growth in relation to peer and romantic rejection.

Building upon Raymaekers et al. (2020), our findings indicate that accepting one's illness has social benefits, including encouraging CD disclosure and reducing fears of stigmatization. Higher peer support correlated with greater illness acceptance, with illness acceptance itself associated with fewer romantic rejection concerns, confirming the importance of acceptance for healthy interpersonal relationships (O'Donnell and Habenicht, 2022). Our results also reaffirm peer support's vital role in anxieties among adolescents with CD (Lehardy and Fowers, 2020; Letkiewicz et al., 2023). These findings are also consistent with recent studies showing that people with low self-esteem are more motivated to establish social relationships for self-protection (Cameron et al., 2010), and therefore they view indirect support seeking as an appealing strategy, which seemingly enables them to garner support and enhance social connectedness (e.g., Cacioppo et al., 2010).

The enrichment dimension of illness identity was not found to play a significant role in romantic rejection concerns. These results align with Raymaekers et al. (2020) and O'Donnell and Habenicht (2022), indicating that illness acceptance, not enrichment, predicts romantic rejection concerns. This suggests acceptance as a more fundamental step in illness integration. Future research could explore enrichment's role in CD adjustment beyond acceptance.

Although the paper focused on concerns of romantic rejection, preferences for romantic partner with CD emerged as a distinguished dimension of our measuring instruments, and results of the correlational analysis can help explaining when a preference for partner who shares the illness may emerge and why. Interestingly, more than a quarter of the participants would prefer a partner also with CD or on a GFD. This proportion is double compared to that found in a recent survey in the US adult population with CD pertaining to preference for a partner who is also on a GFD (Lebovits et al., 2022). Adolescents with lower CDPQOL, experiencing uncertainty, isolation, and limitations due to CD, expressed a higher preference for a romantic partner with CD, presumably for enhanced understanding and empathy. This preference did not correlate significantly with self-esteem or illness identity, implying these factors have limited influence on partner preference. In sum, although the preference for a partner with the same disease might lessen emotional stress and fear of judgment, the complexity of romantic preferences among adolescents seems to extend beyond illness-related factors.

### 4.1 Limitations

This work offers cross-diagnostic insights, illuminating how self-esteem, illness identity, and social support influence adolescents with chronic diseases in coping with peer and romantic rejection, particularly for conditions requiring dietary modifications or associated with autoimmune or gastrointestinal diseases (Lambert and Keogh, 2015). To our knowledge, it is the first to explore romantic rejection concerns among adolescents and young adults with CD, thus expanding knowledge of CD's psychosocial effects.

However, these findings should be interpreted considering some constraints. The cross-sectional design limits our ability to confirm causal or temporal relations between variables, and future longitudinal research should delve into the intricate relationship between self-esteem, illness identity, social support, fear of romantic rejection, and HRQOL, as recommended in diabetes research (Raymaekers et al., 2020). Our non-clinical, convenience sample may not fully represent German adolescents and young adults with CD, as participants, mostly from the national CD society, may be more adjusted than their peers. The interpretation of gender differences in concerns of romantic rejection warrants caution due to our sample's gender imbalance, reflecting the higher prevalence of celiac disease among females (Lebwohl et al., 2018). The limited number of male participants (*n* = 28) constrains our ability to draw robust conclusions for young male patients and perform comprehensive gender comparisons of the relationships examined in this study. Future studies should attempt to recruit a more gender-balanced sample, with a sufficient representation of males, to enable a more accurate analysis of gender similarities or disparities. External validity is also limited due to our homogeneous German sample. Future research should encompass more diverse samples, such as sexual minorities and the LGBT+ community, to understand varying experiences regarding illness and romance. Examining cultural differences by comparing experiences across countries with varying CD awareness and GF options will also enrich the understanding of CD's impact on social-romantic peer relationships.

Our measurement approach also has limitations. The single-item self-esteem measure may not capture its multifaceted nature (Marsh and Craven, 2006), and more comprehensive measures, such as the Celiac Disease Adherence Test (Gładyś et al., 2020), should be considered in future research. Furthermore, our analysis only included two dimensions of illness identity, acceptance, and enrichment, neglecting potential influences of rejection and engulfment. Also, the findings are based on self-reported data without clinical or psychological assessments, leading to potential shared method variance.

Finally, this study did not explore other crucial aspects of romantic relationships like dating behaviors, actual experiences, intimacy, and broader social rejection contexts. Future work could gain deeper insights by using qualitative methods or multidimensional instruments to comprehend vulnerabilities of adolescents with CD in the context of romantic relationships (Balch, 2014). Scales like the Dating Anxiety Scale for Adolescents (Glickman and La Greca, 2004) can be useful in this endeavor. Research should also develop CD-specific instruments capturing unique aspects of romantic relationships for people with CD. Accurate assessment in this context could help prevent future disorders or problematic social and romantic relationships in this population.

### 4.2 Practical implications

Pertaining to practical implications, this study highlights the importance of comprehensive psychosocial support for adolescents with CD (Coburn et al., 2019). Traditional healthcare often overlooks psychological symptoms, focusing instead on dietary adherence and physical symptoms, potentially neglecting important psychosocial factors affecting disease management and quality of life (Wheeler et al., 2022). Thus, we advocate for the integration of psychological services into CD healthcare, aiming to optimize physical and psychosocial health.

Our findings underline the importance of addressing root mechanisms of rejection worries in young CD patients. For those with low self-esteem, increasing peer support could reduce romantic rejection concerns, utilizing varied group-based support methods (Köstlin et al., 2023). Such interventions can boost resilience, reduce loneliness, and enhance social acceptance (Frohlich, 2014; Kulandaivelu and Kohut, 2021). CD-focused activities like summer camps and cost-efficient online peer support also positively influence social support, illness acceptance, and dietary compliance (Köstlin et al., 2023). For high self-esteem adolescents, building a positive illness identity and promoting illness acceptance, possibly through methods like Acceptance and Commitment Therapy (ACT; Hayes et al., 2006), could mitigate romantic rejection concerns and restore positive perceptions about one's own desirability.

Increasing adolescents' knowledge and confidence in disclosing their condition may also improve their confidence in social situation, ultimately enhancing their HRQOL (Wheeler et al., 2022). Enhancing disclosure skills and discussing self-affirming, empowering self-disclosure methods could reduce the perceived negative consequences of revealing their condition. Finally, integrating socioemotional experience and rejection worry inquiries into clinical interactions could foster rapport, facilitate psychology referrals, and encourage development of targeted psychological interventions.

## Data availability statement

The datasets presented in this study can be found in the online OSF repository at the following link: https://osf.io/xsdn2/. Further inquiries can be directed to the corresponding author.

## Ethics statement

The studies involving humans were approved by BIGSSS Ethics Committee, Jacobs University Bremen, Karl-Heinz Ladeur (Chair), April 30, 2020. The studies were conducted in accordance with the local legislation and institutional requirements. Written informed consent for participation in this study was provided by the participants' legal guardians/next of kin.

## Author contributions

MS: Conceptualization, Formal analysis, Investigation, Methodology, Software, Writing – original draft. MZ: Writing – review & editing.

## Footnotes

^1^Although maintaining a GFD while dating presents unique challenges for youth with CD, it is important to stress that research indicates that healthy partners often show resilience and indifference, suggesting that romantic rejection concerns may be largely unfounded (Roy et al., 2016; Jordan et al., 2021). For example, it has been found that partner support in sharing GFD adherence can even boost relationship satisfaction (Alley, 2015). Similarly, Boon and Holmgren (2019) found out that CD posed only slight restrictions on adults' couple-related daily tasks and relationship upkeep activities, with it only seldom causing disputes. Moreover, Aloni et al. (2019) found that individuals adhering to a GFD were viewed with favorable and unfavorable characteristics, including being fastidious, particular, insistent, vocal in their dissatisfaction, and critical, while simultaneously considered health-conscious, self-controlled, empathetic, and vigorous. These perceptions did not universally hinder relationship formation.
