# Supplementary material for: Love beyond gluten: self-esteem, illness identity, and social support in romantic rejection concerns among adolescents with celiac disease
Source: Front Psychol. 2024 May 20;15:1335201. doi: 10.3389/fpsyg.2024.1335201 (PMC11144883; doi:10.3389/fpsyg.2024.1335201)
Supplement: Supplementary file 1 [file Data_Sheet_1.docx]

Supplementary Material

**Title: Love Beyond Gluten: Self-Esteem, Illness Identity, and Social Support in Romantic Rejection Concerns Among Adolescents with Celiac Disease**

**Additional exploratory analysis on the role of Gender in romantic rejection concerns**

The results of our hierarchical regression models suggest that gender was not a significant predictor of romantic rejection concerns. In order to further explicate the relationship between gender and romantic rejection concerns, we repeated the regression analysis with another exploratory model that included interactions between gender (1 = male) and the main predictors, namely illness acceptance, self-esteem, and social support, in predicting romantic rejection concerns. The results indicated that the interaction between gender and illness identity was not significant (B = -0.464, SE = 0.479, p = .336). In addition, the interaction between gender and social support was not significant (B = 0.659, SE = 0.465, p = .159). However, the interaction between gender and self-esteem was significant (B = 0.673, SE = 0.259, p = .011). Follow-up simple slope analysis indicated that that higher self-esteem predicted reduced concerns only among female participants (B = -0.201, SE = 0.097, p < .040) but not among male participants (p = .085).

However, this could have been the result of the relatively small number of male participants in the sample. Accordingly, these exploratory results should be interpreted with caution, and may be used only to stress the need to obtain a larger, more balanced sample of male and female participants in order to learn about the different processes involved in romantic rejection concerns in celiac disease in general and among young patients in particular.

**Table S.1**

*Sample Characteristics for All Sample, By Gender, and By Relationship Status Group*

|  | Total (N=165) |  | Boys  (n=28) | Girls (n=134) | No relationship (n=114) | In relationship (n=51) | Gender comparison  (p value) | Relationship comparison  (p value) |
| --- | --- | --- | --- | --- | --- | --- | --- | --- |
| **Gender** |  |  |  |  |  |  |  | 0.758 |
| Not known/diverse |  |  |  |  | 2 (1.8%) | 1 (2.0%) |  |  |
| Male |  |  |  |  | 21 (18.4%) | 7 (13.7%) |  |  |
| Female |  |  |  |  | 91 (79.8%) | 43 (84.3%) |  |  |
| **Age** |  |  |  |  |  |  | .804 | 0.010 |
| N-Miss | 3 |  | 1 | 2 | 3 | 0 |  |  |
| Mean (SD) | 17.179 (2.499) |  | 16.889 (2.708) | 17.235 (2.486) | 16.838 (2.376) | 17.922 (2.622) |  |  |
| Range | 14.000 - 25.000 |  | 14.000 - 22.000 | 14.000 - 25.000 | 14.000 - 22.000 | 14.000 - 25.000 |  |  |
| **Socioeconomic status** |  |  |  |  |  |  | .212 | 0.420 |
| N-Miss | 1 |  | 0 | 1 | 1 | 0 |  |  |
| Mean (SD) | 3.299 (0.675) |  | 3.464 (0.744) | 3.256 (0.659) | 3.327 (0.619) | 3.235 (0.790) |  |  |
| Range | 1.000 - 5.000 |  | 2.000 - 5.000 | 1.000 - 5.000 | 2.000 - 5.000 | 1.000 - 5.000 |  |  |
| **Romantic relationship status** |  |  |  |  |  |  | .758 |  |
| Not in relationship | 114 (69.1%) |  | 21 (75.0%) | 91 (67.9%) |  |  |  |  |
| In relationship | 51 (30.9%) |  | 7 (25.0%) | 43 (32.1%) |  |  |  |  |
| **Age of CD diagnosis** |  |  |  |  |  |  | .121 | .881 |
| N-Miss | 16 |  | 2 | 14 | 11 | 5 |  |  |
| Mean (SD) | 9.490 (5.264) |  | 10.654 (5.215) | 9.367 (5.250) | 9.447 (4.986) | 9.587 (5.898) |  |  |
| Range | 0.000 - 21.000 |  | 1.000 - 21.000 | 0.000 - 21.000 | 1.000 - 21.000 | 0.000 - 21.000 |  |  |
| **No. years living with CD** |  |  |  |  |  |  | .062 | .493 |
| N-Miss | 19 |  | 3 | 16 | 14 | 5 |  |  |
| Mean (SD) | 7.685 (5.044) |  | 6.240 (4.275) | 7.856 (5.145) | 7.490 (4.719) | 8.109 (5.720) |  |  |
| Range | 0.000 - 22.000 |  | 0.000 - 16.000 | 0.000 - 22.000 | 0.000 - 20.000 | 0.000 - 22.000 |  |  |
| **Level of GFD adherence** |  |  |  |  |  |  | .599 | .070 |
| Mean (SD) | 9.548 (0.974) |  | 9.643 (0.591) | 9.519 (1.045) | 9.640 (0.808) | 9.343 (1.255) |  |  |
| Range | 2.000 - 10.000 |  | 7.500 - 10.000 | 2.000 - 10.000 | 4.500 - 10.000 | 2.000 - 10.000 |  |  |
| **Other food intolerances/Allergies** |  |  |  |  |  |  | .889 | .064 |
| N-Miss | 4 |  | 0 | 4 | 3 | 1 |  |  |
| No | 96 (59.6%) |  | 16 (57.1%) | 78 (60.0%) | 72 (64.9%) | 24 (48.0%) |  |  |
| Have additional mild allergies | 38 (23.6%) |  | 6 (21.4%) | 31 (23.8%) | 25 (22.5%) | 13 (26.0%) |  |  |
| Have additional strong allergies | 27 (16.8%) |  | 6 (21.4%) | 21 (16.2%) | 14 (12.6%) | 13 (26.0%) |  |  |
| **Other Illnesses/ medical conditions** |  |  |  |  |  |  | .570 | .857 |
| N-Miss | 44 |  | 8 | 36 | 31 | 13 |  |  |
| No other medical conditions | 75 (62.0%) |  | 13 (65.0%) | 61 (62.2%) | 51 (61.4%) | 24 (63.2%) |  |  |
| Other medical conditions | 46 (38.0%) |  | 7 (35.0%) | 37 (37.8%) | 32 (38.6%) | 14 (36.8%) |  |  |
| **Celiac symptoms** |  |  |  |  |  |  | .348 | .201 |
| Not sure/ don’t know | 20 (12.1%) |  | 3 (10.7%) | 17 (12.7%) | 15 (13.2%) | 5 (9.8%) |  |  |
| No symptoms | 26 (15.8%) |  | 7 (25.0%) | 19 (14.2%) | 22 (19.3%) | 4 (7.8%) |  |  |
| Moderate symptoms | 52 (31.5%) |  | 9 (32.1%) | 43 (32.1%) | 35 (30.7%) | 17 (33.3%) |  |  |
| Strong symptoms | 67 (40.6%) |  | 9 (32.1%) | 55 (41.0%) | 42 (36.8%) | 25 (49.0%) |  |  |

*Note*. N-Miss = Number of missing values. Continuous variables are summarized by Means (SDs), Minimum-Maximum values with ANOVA (t-test with equal variances) p-values. Categorical variables are summarized by count (column percent) with a chi-square p-values.

**Table S.2**

*Results of Principal Component Analysis: Romantic Rejection Items*

|  | All items |  | Without item 7 |  |
| --- | --- | --- | --- | --- |
| Item | Component 1: Romantic rejection worries | Component 2: Romantic preference | Component 1 | Component 2 |
| 1. I am worried that a boy/girl will not want to date me because I have celiac | **0.970** | -0.093 | **0.950** | -0.045 |
| 2. I sometimes worry that I will be hard for me to find a girlfriend/boyfriend because I have celiac | **0.947** | -0.075 | **0.880** | 0.053 |
| 3. I am afraid that boys/girls like will see my celiac as a problem | **0.871** | 0.034 | **0.925** | -0.059 |
| 4. When I date someone, I want them to know nothing about my celiac disease. | **0.769** | 0.148 | 0.316 | 0.161 |
| 5. I sometimes worry that no one will want to be with me because I have celiac | -0.057 | 1.023 | **0.770** | 0.114 |
| 6. I would prefer a partner that also has celiac | 0.257 | 0.064 | -0.067 | **0.606** |
| 7. I would prefer a partner that does not have celiac | -0.023 | 0.212 |  |  |
| 8. It doesn't matter to me if my partner has celiac or not | -0.303 | -0.036 | 0.043 | **-0.623** |
| Eigenvalue | 3.865 | 1.141 | 3.853 | 1.093 |
| % variance explained | 41.9% | 14.2% | 46.2% | 11.5% |

**Table S.3**

*Results of Confirmatory Factor Analysis for Illness Identity and CDPQOL*

| Model | Scaled χ^2^(df) | SCF | Δχ2  (df) | AIC | RMSEA [90% CI] | CFI | TLI | SRMR | CRs | AVEs | Modifications |
| --- | --- | --- | --- | --- | --- | --- | --- | --- | --- | --- | --- |
| Illness identity: two factors | 135.902(53)*** | 1.152 |  | 5351.366 | .099 [.080, .118] | .893 | .867 | .063 | >=.650 | >=.509 |  |
| Illness identity: two factors (revised) | 74.652(51)* | 1.120 | 69.957(2)*** | 5282.418 | .057 [.025, .083] | .970 | .962 | .053 | >=.632 | >=.497 | Residual covariances between items 1 and 2, between items 6 and 8 |
| CDPQOL: four factors | 175.812(113)*** | 1.045 |  | 7822.005 | .061 [.043, .078] | .930 | .916 | .059 | >=.358 | >=.360 |  |
| CDPQOL: four factors (revised) | 151.204(110)** | 1.043 | 21.444(3)*** | 7801.917 | .050 [.028, .069] | .954 | .944 | .058 | >=.358 | >=.360 | Residual covariances between items 1 and 3, 13 and 15, and 15 and 18 |

Note. CDPQOL = Celiac Disease Pediatric Quality of Life. SCF = Scaling correction Factor. AIC = Akaike information criterion; BIC = Bayesian information criterion; CI = confidence interval; CFI = comparative fit index; DF = degrees of freedom; RMSEA = root mean square error of approximation; SRMR = standardized root mean squared residual; TLI = Tucker–Lewis index; CR = composite reliability; AVE = average variance extracted. Chi-square values and difference test based on robust standard errors.

* p < .05, ** p < .01, *** p < .001.

**Table S.4**

*Means (SDs) for All Items Measuring Romantic Rejection Concerns and Preferences in All Sample and by Gender and Relationship Status With Results of Difference Test*

| Romantic rejection item | All sample (N = 165) | Girls (n=134) | Boys  (n = 28) | t-test (df), p value | Not in relationship (n=114) | In relationship (n=51) | t-test (df), p value |
| --- | --- | --- | --- | --- | --- | --- | --- |
|  |  |  |  |  |  |  |  |
| 1. I am worried that a boy/girl will not want to date me because I have celiac | 2.18 (1.42) | 2.17 (1.43) | 2.18 (1.36) | t(159) = -0.05, p = .96 | 2.38 (1.51) | 1.75 (1.07) | t(132.34) = 3.07, = .003 |
| 2. I sometimes worry that I will be hard for me to find a girlfriend/boyfriend because I have celiac | 2.44 (1.43) | 2.40 (1.44) | 2.57 (1.35) | t(160) = -0.57, p = .57 | 2.62 (1.46) | 2.02 (1.27) | t(109.41) = 2.69, p = .008 |
| 3. I am afraid that boys/girls like will see my celiac as a problem | 2.54 (1.45) | 2.56 (1.48) | 2.46 (1.29) | t(159) = 0.31, p = .76 | 2.67 (1.50) | 2.24 (1.32) | t(108.51) = 1.88, p = .06 |
| 4. I sometimes worry that no one will want to be with me because I have celiac | 2.02 (1.33) | 2.01 (1.33) | 2.04 (1.32) | t(159) = -0.10, p = .92 | 2.13 (1.35) | 1.78 (1.27) | t(102.36) = 1.59, p = .11 |
| 5. When I ask someone on a date, I would prefer he/she not to know that I have celiac | 1.92 (1.25) | 1.90 (1.23) | 1.96 (1.32) | t(160) = -0.24, p = .81 | 1.95 (1.30) | 1.86 (1.17) | t(163) = 0.40, p = .69 |
| 6. I would prefer a partner that also has celiac | 2.60 (1.41) | 2.59 (1.38) | 2.71 (1.56) | t(160) = -0.42, p = .67 | 2.59 (1.40) | 2.63 (1.46) | t(163) = -0.17, p = .87 |
| 7. It doesn't matter to me if my partner has celiac or not | 4.22 (1.14) | 4.30 (1.05) | 3.78 (1.48) | t(31.48) = 1.75, p = .09 | 4.17 (1.17) | 4.33 (1.05) | t(162) = -0.86, p = .39 |
| Romantic concerns scale | 2.22 (1.16) | 2.21 (1.14) | 2.24 (1.19) | t(159) = -0.15, p = .88 | 2.35 (1.20) | 1.93 (1.02) | t(162) = 2.18, p = .03 |
| Romantic preference scale | 2.19 (1.05) | 2.15 (1.00) | 2.46 (1.25) | T(159) = -1.44, p = .15 | 2.21 (1.04) | 2.15 (1.08) | t(162) = 0.34, p = .73 |

**Table S.5**

*Results of Hierarchical Linear Regression Predicting CDPQOL*

| Predictors | CDPQOL-Social | | | | CDPQOL-Uncertainty | | | | CDPQOL-Isolation | | | | CDPQOL-Limitation | | | | CDPQOL-Total | | | |
| --- | --- | --- | --- | --- | --- | --- | --- | --- | --- | --- | --- | --- | --- | --- | --- | --- | --- | --- | --- | --- |
| *Predictors* | *Estimates* | *std. Error* | *std. Beta* | *p* | *Estimates* | *std. Error* | *std. Beta* | *p* | *Estimates* | *std. Error* | *std. Beta* | *p* | *Estimates* | *std. Error* | *std. Beta* | *p* | *Estimates* | *std. Error* | *std. Beta* | *p* |
| (Intercept) | 6.480 | 15.118 | 0.000 | 0.669 | 5.190 | 19.352 | -0.000 | 0.789 | 1.179 | 15.147 | 0.000 | 0.938 | -0.411 | 18.312 | 0.000 | 0.982 | 1.781 | 12.869 | 0.000 | 0.890 |
| 1=male | 6.561 | 3.702 | 0.122 | 0.079 | -3.227 | 4.920 | -0.049 | 0.513 | 7.888 | 3.690 | 0.144 | **0.034** | 1.481 | 4.484 | 0.023 | 0.742 | 4.105 | 3.255 | 0.083 | 0.210 |
| Years since diagnosis | 0.146 | 0.301 | 0.036 | 0.630 | -0.127 | 0.391 | -0.026 | 0.746 | 0.146 | 0.302 | 0.035 | 0.630 | -0.306 | 0.365 | -0.062 | 0.403 | 0.036 | 0.260 | 0.010 | 0.890 |
| 1 = In a relationship | -3.496 | 3.125 | -0.079 | 0.265 | -7.924 | 4.145 | -0.149 | 0.058 | -5.554 | 3.120 | -0.124 | 0.077 | -0.448 | 3.786 | -0.008 | 0.906 | -4.110 | 2.748 | -0.102 | 0.137 |
| Age | 0.662 | 0.595 | 0.080 | 0.268 | 1.062 | 0.768 | 0.107 | 0.169 | 1.418 | 0.596 | 0.167 | **0.019** | 1.538 | 0.721 | 0.154 | **0.035** | 1.192 | 0.511 | 0.158 | **0.021** |
| Socioeconomic status | 2.083 | 2.334 | 0.063 | 0.374 | 3.584 | 2.999 | 0.091 | 0.234 | -1.546 | 2.331 | -0.046 | 0.508 | 1.521 | 2.828 | 0.038 | 0.592 | 1.361 | 1.989 | 0.046 | 0.495 |
| Peer support | 10.295 | 2.148 | 0.387 | **<0.001** | 7.816 | 2.761 | 0.245 | **0.005** | 9.671 | 2.139 | 0.358 | **<0.001** | 13.928 | 2.602 | 0.438 | **<0.001** | 10.197 | 1.824 | 0.422 | **<0.001** |
| Illness identity: Acceptance | 4.576 | 2.167 | 0.183 | **0.037** | 7.294 | 2.798 | 0.241 | **0.010** | 5.279 | 2.177 | 0.207 | **0.017** | 0.912 | 2.625 | 0.030 | 0.729 | 4.402 | 1.865 | 0.192 | **0.020** |
| Illness identity: Enrichment | -3.047 | 1.566 | -0.144 | 0.054 | -0.945 | 2.068 | -0.037 | 0.648 | 1.466 | 1.564 | 0.068 | 0.351 | -0.661 | 1.896 | -0.026 | 0.728 | -1.053 | 1.370 | -0.054 | 0.444 |
| Romantic rejection concerns | -3.379 | 1.181 | -0.214 | **0.005** | -4.841 | 1.529 | -0.255 | **0.002** | -2.947 | 1.185 | -0.183 | **0.014** | -4.545 | 1.431 | -0.240 | **0.002** | -3.604 | 1.019 | -0.251 | **0.001** |
| Observations | 139 | | | | 133 | | | | 138 | | | | 139 | | | | 132 | | | |
| R^2^ / R^2^ adjusted | 0.408 / 0.367 | | | | 0.338 / 0.289 | | | | 0.435 / 0.396 | | | | 0.394 / 0.352 | | | | 0.499 / 0.462 | | | |

*Note*. CDPQOL = Celiac Disease Pediatric Quality of Life.

**Figure S.1**

*Distribution (Percentages) of Items Measuring Celiac-Related Romantic Concerns and Preferences In the Entire Sample*

*Note*. For ease of interpretation, responses on a 5-point Likert scale ranging from 1 (strongly disagree) to 5 (strongly agree) were categorized into three groups: disagreement (response options 1 and 2), neutrality (response option 3), and agreement (response options 4 and 5).
